# A Bifunctional Fibrous Scaffold Implanted with Amorphous Co_2_P as both Cathodic and Anodic Stabilizer for High‐Performance Li─S Batteries

**DOI:** 10.1002/advs.202501153

**Published:** 2025-04-01

**Authors:** Gang Zhao, Tianran Yan, Lei Wang, Cheng Yuan, Tong Chen, Bin Wang, Chen Cheng, Pan Zeng, Yude Su, Liang Zhang

**Affiliations:** ^1^ Institute of Functional Nano & Soft Materials (FUNSOM) Soochow University 199 Ren'ai Road Suzhou Jiangsu 215123 China; ^2^ School of Chemistry and Materials Science University of Science and Technology of China Hefei Anhui 230026 China; ^3^ China Minmetals Graphite Industry Co., Ltd. (Heilongjiang) Hegang Heilongjiang 154100 China; ^4^ Institute for Advanced Study School of Mechanical Engineering Chengdu University Chengdu 610106 China; ^5^ Jiangsu Key Laboratory of Advanced Negative Carbon Technologies Soochow University Suzhou Jiangsu 215123 China

**Keywords:** amorphous structure, catalytic conversion, Li dendrites, Li–S batteries, shuttle effect

## Abstract

The shuttling of lithium polysulfides (LiPSs) and the formation of lithium dendrites have substantially impeded the practical application of lithium–sulfur (Li─S) batteries. To simultaneously solve these issues, a porous carbon fibrous scaffold embedded with amorphous Co_2_P (A─Co_2_P) is designed as both a cathodic and anodic stabilizer to construct high‐rate and long‐life Li─S batteries. The meticulously designed self‐supporting membrane with an integrated carbon network and porous structure offers superior conductivity and copious spaces for uniform Li_2_S precipitation in the cathode and Li deposition in the anode. Moreover, the incorporated A─Co_2_P provides abundant unsaturated sites, which can not only facilitate the exposure of active sites but also modulate the electronic configuration for enhanced LiPSs adsorption and catalysis capability. Concurrently, the presence of lithiophilic A─Co_2_P sites also reinforces the stability of Li anode with the suppressed formation of dendrites. The constructed full Li─S batteries deliver a high areal capacity of 6.6 mAh cm^−2^ with a sulfur loading of 8.5 mg cm^−2^ and a low capacity decay rate of 0.047% per cycle after 800 cycles. This work provides a simple yet effective strategy to construct practical Li─S batteries by simultaneously addressing LiPSs shuttling and Li dendrite growth.

## Graphical abstract

An amorphous Co_2_P‐embedded porous carbon nanofiber membrane (A─Co_2_P/PCNF) via electrospinning/in situ phosphorization is developed to serve as the dual‐electrode stabilizer for Li─S batteries. The amorphization strategy enables more exposed undercoordinated Co sites with optimized *d*‐band centers, enhanced LiPSs adsorption/catalysis, and lithiophilicity‐guided uniform Li deposition, leading to an areal capacity of 6.6 mAh cm⁻^2^ and a mere 0.047% decay/cycle over 800 cycles.

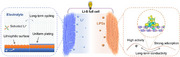

## 1 Introduction

Lithium─sulfur (Li─S) batteries have been considered as one of the most promising energy storage systems because of their high theoretical specific capacity (1675 mAh g^−1^), low cost, and environmental benignity.^[^ ^1^, ^2^, ^3^ ^]^ Nevertheless, there are a plethora of issues associated with the use of monolithic S as the cathode and Li metal as the anode, which significantly impedes the practical application of Li─S batteries.^[^ ^4^ ^]^ More specifically, the insulating nature of S and the severe volume change during the cycling process result in insufficient utilization of active substances and instability of electrodes, especially under high sulfur loading conditions.^[^ ^5^ ^]^ In addition, the notorious shuttle effect of intermediate lithium polysulfides (LiPSs) not only results in further loss of active substances but also corrodes the anodes to form dead Li, leading to fast capacity decay and inferior cycle life.^[^ ^6^, ^7^, ^8^ ^]^ Concurrently, the use of Li metal as the anode also presents significant safety concerns, with the most critical of these being the formation of Li dendrites due to the uneven deposition of Li during the charge‐discharge cycling,^[^ ^9^, ^10^, ^11^ ^]^ which can result in puncturing of the separators in severe cases and lead to the short circuit of the batteries.^[^ ^12^, ^13^ ^]^

So far, different strategies have been proposed to address the aforementioned issues. For example, extensive efforts have been devoted to the rational design of sulfur substrates with high porosity and good conductivity to enhance the utilization of active materials.^[^ ^5^, ^14^ ^]^ To further inhibit the shuttle effect, numerous metal‐based electrocatalysts have been developed to chemically adsorb LiPSs and enhance their redox kinetics.^[^ ^15^, ^16^, ^17^ ^]^ In addition, various strategies have been developed to stabilize the Li anode, including the preparation of Li host materials with lithiophilic surfaces, electrolyte modification, and construction of artificial solid electrolyte interface (SEI) layers.^[^ ^10^, ^11^, ^18^, ^19^ ^]^ Among them, one straightforward and efficacious strategy for enhancing the Li anode performance is coating the Li metal surface with an artificial lithiophilic layer to regulate the Li^+^ flux and prevent the separator from being pierced by Li dendrites.^[^ ^20^, ^21^ ^]^ For example, a “two‐in‐one” strategy has recently been proposed to construct a host matrix comprising abundant lithiophilic/sulfiphilic sites and rational ordered spatial structure to promote sulfur redox kinetics and facilitate uniform Li deposition simultaneously. In addition, Yu and coworkers reported a carbon fiber network embedded with TiN‐VN as a potential stabilizer for both the S cathode and Li anode in Li─S batteries.^[^ ^12^ ^]^ As the cathode host, TiN‐VN@CNFs demonstrate the synergistic anchoring and catalyzing ability for LiPSs to mitigate the shuttle effect. Meanwhile, the well‐designed matrix with lithiophilic features can facilitate the homogeneous Li deposition for assuaging Li dendrite formation. Furthermore, electrocatalysts including V_2_C MXene,^[^ ^22^ ^]^ CoSe@BNCNTs/CC,^[^ ^23^ ^]^ and V_8_C_7_─VO_2_^[^ ^24^ ^]^ have also been reported as dual functional stabilizers for Li─S batteries. However, it is worth mentioning that these materials lack either the requisite porous structure to accommodate active materials or the continuous architecture to provide robust stability and long‐range conductivity, thereby rendering the difficult to meet the commercial requirements of Li─S batteries.

Notably, the development of advanced electrocatalysts stands as a cornerstone in overcoming the intrinsic limitations of Li─S batteries, particularly the notorious polysulfide shuttle effect and uncontrolled lithium dendrite formation that severely compromise cycling stability and safety. Within the landscape of transition metal‐based electrocatalysts, metal phosphides have garnered significant attention owing to their inherently tunable electronic structures and multifunctional catalytic capabilities. Their superior electrical conductivity enables efficient charge transfer during sulfur reduction/oxidation reactions, while the polarized M─P (metal‐phosphorus) bonds exhibit strong Lewis acidity for chemically anchoring LiPSs via M─S interactions.^[^ ^25^, ^26^, ^27^ ^]^ Beyond cathode stabilization, a paradigm shift occurred with the discovery of lithiophilic characteristics in metal phosphides,^[^ ^28^ ^]^ where their surface electronic states enable homogeneous Li^+^ flux regulation and lower nucleation barriers, thereby suppressing dendrite proliferation at the anode. However, conventional crystalline phosphides suffer from limited active site exposure due to rigid lattice constraints, whereas amorphous counterparts with disordered atomic networks offer unique advantages: 1) high‐entropy atomic configurations generate undercoordinated metal centers with optimized *d*‐band positions near the Fermi level, enhancing both LiPSs conversion kinetics and Li nucleation uniformity, 2) electron delocalization across disordered networks promotes interfacial charge transfer.^[^ ^27^, ^29^ ^]^ Amorphous phosphides offer multiple advantages that sharply contrast with current research trends focusing solely on crystalline materials for electrode modification. Their ability to cooperatively stabilize both S and Li electrodes through synergistic catalytic effects remains an overlooked aspect in advancing Li─S batteries.

In this work, we report the development of a porous carbon fibrous membrane loaded with amorphous Co_2_P (A─Co_2_P/PCNF) achieved through electrospinning and in situ phosphorization, which has been applied as both cathodic and anodic stabilizer for high‐performance Li─S batteries. We demonstrate that such an amorphization strategy can effectively expose the Co‐active sites containing unsaturated coordination sites and modulate its *d* band center closer to the Fermi level, which considerably enhances the LiPSs adsorption/catalysis and uniform Li deposition with the suppression of Li dendrites. Meanwhile, the 1D porous nanofibers with abundant lithiophilic/sulfiphilic sites provide good electronic and ionic conductivity as well as copious space for uniform Li_2_S precipitation and Li deposition during the charge‐discharge cycles. Benefiting from the synergistic merits, the assembled full Li─S batteries deliver a high areal capacity of 6.6 mAh cm^−2^ at 0.1 C under a high sulfur loading of 8.5 mg cm^−2^ and achieves a high cycling stability with a mere 0.047% capacity decay per cycle after 800 cycles at 1 C. Our present study provides a promising strategy to improve the electrochemical performance of Li─S batteries toward practical application by simultaneously improving LiPSs retention/catalysis and suppressing Li dendrite growth.

## 2 Results and Discussion

**Figure 1:**
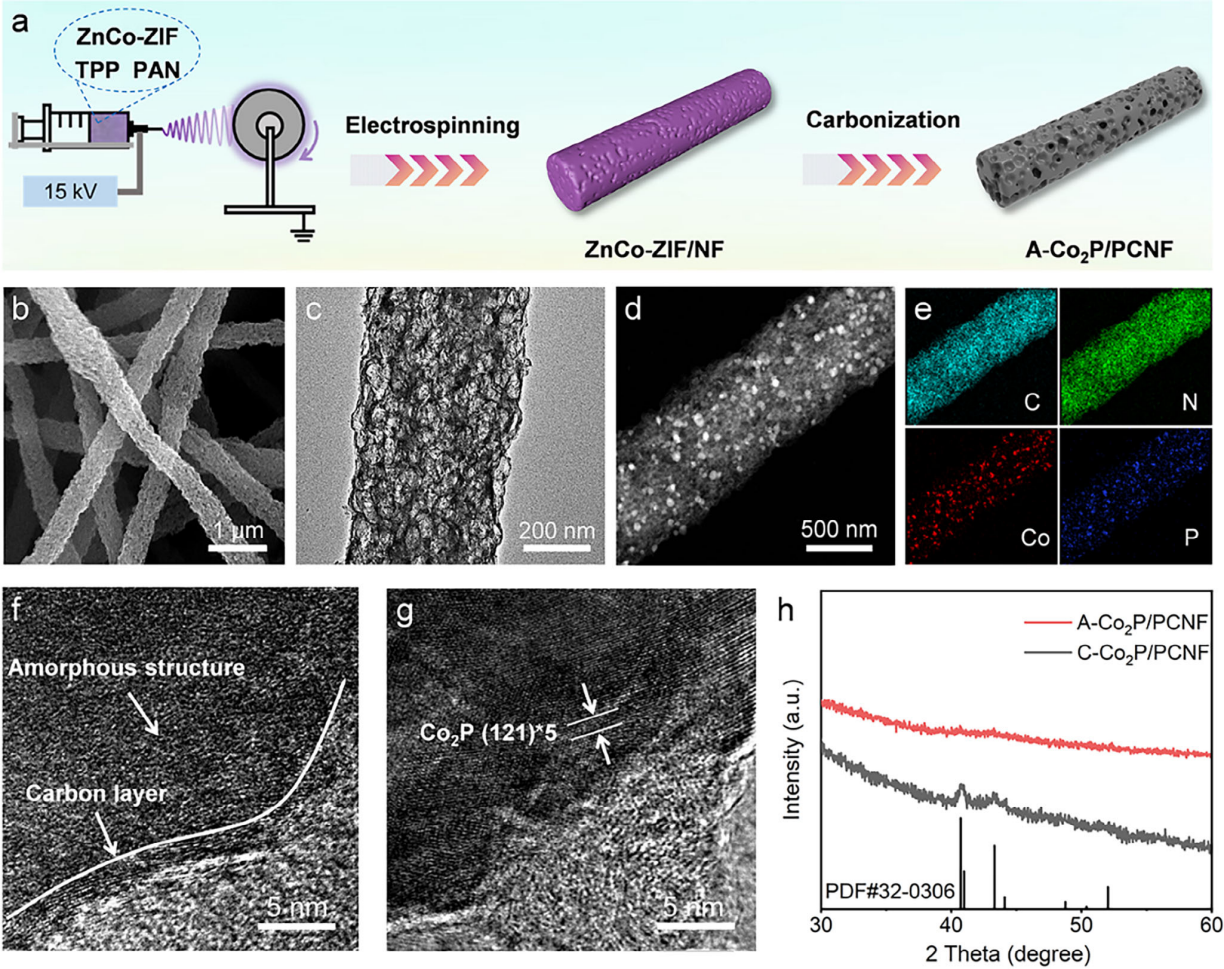
a) Schematic illustration of the synthetic procedure of A─Co_2_P/PCNF. b) SEM image of A─Co_2_P/PCNF. c) TEM image of A─Co_2_P/PCNF. d,e) HAADF‐STEM image and corresponding elemental mapping images of A─Co_2_P/PCNF. f) HRTEM image of A─Co_2_P/PCNF. g) HRTEM image of C─Co_2_P/PCNF. h) XRD patterns of A─Co_2_P/PCNF and C─Co_2_P/PCNF.

The synthetic process of A─Co_2_P/PCNF is schematically illustrated in **Figure** **1**a. In general, a (Co, Zn)‐coordinated zeolitic imidazolate framework (CoZn‐ZIF) with a diameter of 200 nm was first synthesized.^[^ ^20^ ^]^ Then, the resulting ZnCo‐ZIF was dispersed in a dimethylformamide solution with a certain amount of triphenylphosphine (PPh_3_) and polyacrylonitrile (PAN) for the following electrospinning process. The thus‐derived fibrous network was subjected to thermal pyrolysis to derive A─Co_2_P/PCNF. During the carbonization process, the Co^2+^ in CoZn‐ZIF was first reduced and aggregated into metallic Co at a relatively low temperature and then underwent an Ullmann‐type reaction to yield cobalt phosphide (4Co + 2PPh_3_ → 2Co_2_P + 3Ph‐Ph).^[^ ^30^, ^31^ ^]^ We deduce that the decomposition of organic linkers of PAN into nitrogen and cyano fragments could form a local reducing atmosphere and induce the amorphization of Co_2_P at high temperatures. Therefore, crystalline Co_2_P (C─Co_2_P) embedded porous carbon nanofiber (C─Co_2_P/PCNF) was also synthesized as a contrast sample by lowering phosphorization temperature.

Scanning electron microscopy (SEM) and transmission electron microscopy (TEM) were employed to analyze the morphological and structural features of A─Co_2_P/PCNF. As illustrated in Figure 1b, A─Co_2_P/PCNF exhibits a remarkably high length‐to‐diameter ratio with a 3D interconnected network structure consisting of 1D hierarchical porous nanofibers. The spontaneously evolved multilevel porosity stems from the aggregation and self‐evaporation of Zn species at high temperatures.^[^ ^32^, ^33^ ^]^ Brunauer–Emmett–Teller analysis further confirms that A─Co₂P/PCNF possesses a high specific surface area of 667 m^2^ g⁻¹, with an average pore diameter of ≈4 nm (Figure S1, Supporting Information). The high porosity of A─Co_2_P/PCNF is expected to provide effective nanoconfinement and uniform distribution for active materials to increase their utilization, while the densely packed carbon nanofibers promise excellent long‐range conductivity for rapid LiPSs conversion. TEM images (Figure 1c,d) further reveal that the 1D porous structure of A─Co_2_P/PCNF is uniformly dispersed with amorphous Co_2_P (A─Co_2_P) nanoparticles. The corresponding energy dispersive X‐ray spectrometry elemental mapping images (Figure 1e) show the homogeneous distribution of C, N, Co, and P in the nanofibers. Moreover, Co and P elements distribute in the same position with a molar ratio of approximately 2:1, manifesting the successful synthesis of Co_2_P. Notably, similar morphology and elemental mapping images are also observed for C─Co_2_P/PCNF (Figures S2, S3, Supporting Information).

High‐resolution TEM (HRTEM) image was recorded to identify the atomic structure of A─Co_2_P/PCNF. As displayed in Figure 1f, A─Co_2_P/PCNF exhibits randomly distributed atoms with no continuous crystal lattice stripes, indicating the lack of long‐range ordered atomic structure. In contrast, C─Co_2_P/PCNF shows aligned lattice fringes with an interlayer separation of 0.23 nm (Figure 1g), corresponding to (121) crystalline planes of orthorhombic Co_2_P. Moreover, the characteristic peaks in the X‐ray diffraction (XRD) patterns (Figure 1h) are ascribed to Co_2_P (PDF# 32–0306). In sharp contrast, the absence of diffraction features in the XRD patterns of A─Co_2_P/PCNF further corroborates that it is amorphous, in good accordance with the HRTEM results.

**Figure 2:**
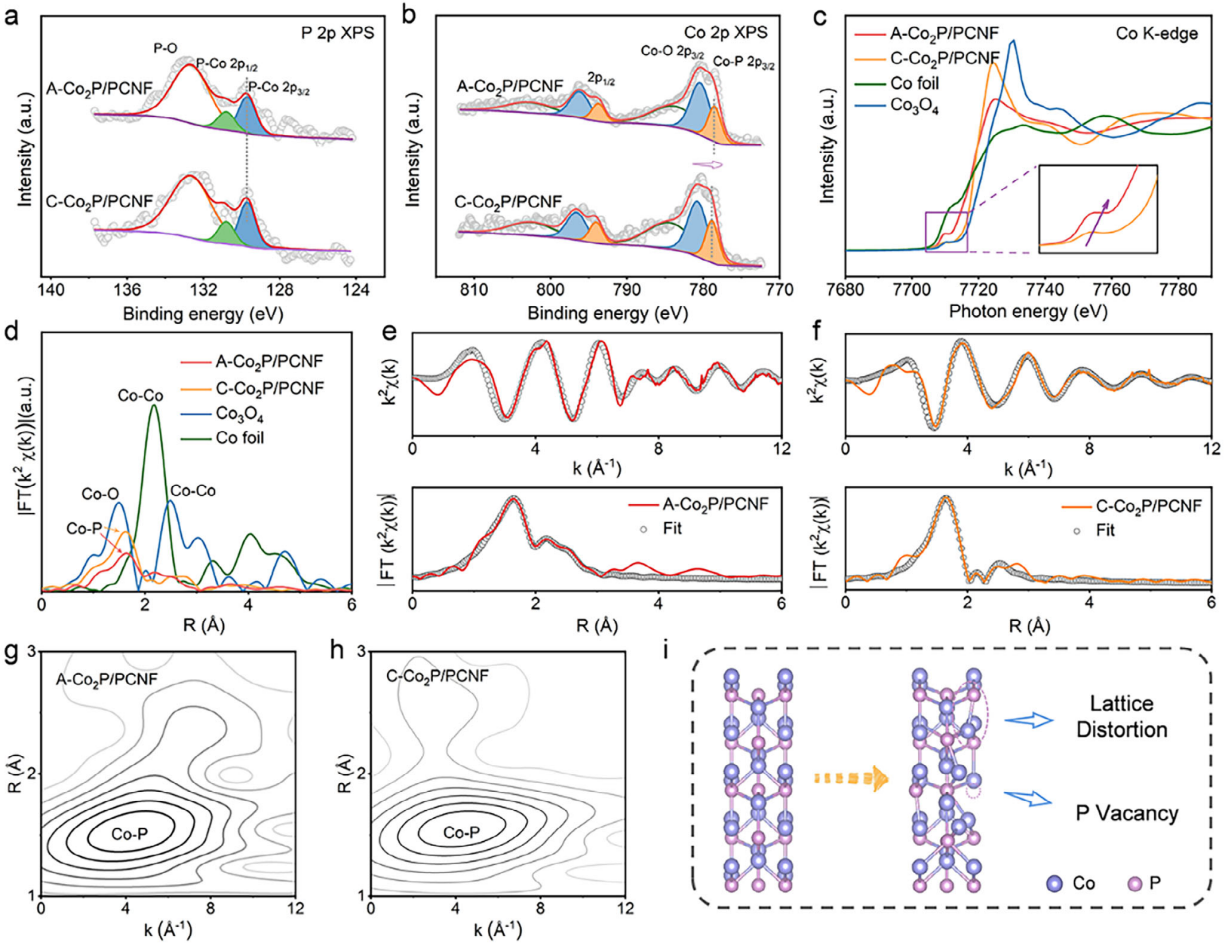
a) P 2p and b) Co 2p XPS spectra of A─Co_2_P/PCNF and C─Co_2_P/PCNF. c) Co K‐edge XANES spectra and d) FT‐EXAFS spectra of A─Co_2_P/PCNF and C─Co_2_P/PCNF. EXAFS fitting curves in k‐space and R‐space of e) A─Co_2_P/PCNF and f) C─Co_2_P/PCNF. WT‐EXAFS plots of g) A─Co_2_P/PCNF and h) C─Co_2_P/PCNF. i) Schematic illustration of crystal structures for A─Co_2_P/PCNF and C─Co_2_P/PCNF.

X‐ray photoelectron spectroscopy (XPS) was carried out to investigate the surface chemical states of A─Co_2_P/PCNF and C─Co_2_P/PCNF. As shown in **Figure** **2**a, the P 2p XPS spectrum of A─Co_2_P/PCNF shows two peaks of P─Co bonds located at 129.8 and 130.8 eV as well as one broad peak of P─O bond located at 134.6 eV,^[^ ^27^ ^]^ which are consistent with the spectrum of C─Co_2_P/PCNF. Correspondingly, the Co 2p XPS spectrum exhibits the two peaks at 778.6 and 793.8 eV that are indexed to the Co 2p_3/2_ and Co 2p_1/2_ of Co─P bond, while the peaks located at 780.5 and 796.3 eV are assigned to the Co 2p_3/2_ and Co 2p_1/2_ of oxidized Co species, respectively. In contrast, the two broad peaks at 784 and 802.8 eV are attributed to the satellite features (Figure 2b).^[^ ^34^, ^35^ ^]^ Interestingly, the peak of Co─P bond for A─Co_2_P/PCNF shifts to lower binding energies compared to that of C─Co_2_P/PCNF, suggesting the lower valence state of Co in A─Co_2_P/PCNF as a consequence of the abundant unsaturated Co species.

To further investigate the electronic state and coordination environment difference between A─Co_2_P/PCNF and C─Co_2_P/PCNF, we measured Co K‐edge X‐ray absorption fine structure (XAFS) spectra. The absorption edges of Co K‐edge X‐ray absorption near‐edge structure (XANES) spectra of A─Co_2_P/PCNF and C─Co_2_P/PCNF are located between that of Co foil and Co_3_O_4_ (Figure 2c), which confirms the oxidation state of Co in both samples. Moreover, the absorption edge of A─Co_2_P/PCNF negatively shifts compared with that of C─Co_2_P/PCNF, indicating the lower Co valence state for the former, as consistent with the above XPS analysis. The enlarged spectra in Figure 2c reveal that A─Co_2_P/PCNF has stronger pre‐edge peaks, which should be ascribed to the increased lattice distortion induced by amorphization.^[^ ^36^, ^37^ ^]^ The corresponding Fourier‐transformed (FT) *k* ^2^‐weighted extended X‐ray absorption fine structure (EXAFS) spectra (Figure 2d) primarily show only one prominent peak located at ≈1.8 Å attributed to Co─P bond coordination. Besides, the wavelet transform (WT) EXAFS analysis (Figure 2g,h; Figure S4, Supporting Information) with powerful resolution in both *k* and *R* spaces also suggests that A─Co_2_P/PCNF (Figure 2g) presents one WT‐maxima at 4.2 Å^−1^, as similar to that of C─Co_2_P/PCNF. The precise coordination environment of Co atoms in C─Co_2_P/PCNF was further unveiled by least‐squares EXAFS fitting. As shown in Figure 2e,f, the fitting curves match well with the experimental spectra in both *k* and *R* spaces, suggesting the high reliability of the fitting results. Furthermore, the quantitative EXAFS fitting results (Table S1, Supporting Information) reveal that the coordination numbers of Co─P and Co─Co bonds are changed from 4.12 and 1.83 for C─Co_2_P/PCNF to 3.83 and 1.96 for A─Co_2_P/PCNF, respectively, which strongly corroborate the unsaturated coordination characteristics of Co atoms in A─Co_2_P/PCNF. Based on the above structural analysis, the theoretical model of A─Co_2_P can be optimized and established (Figure 2i), which contains considerable P vacancy and lattice distortion compared with C─Co_2_P.

**Figure 3:**
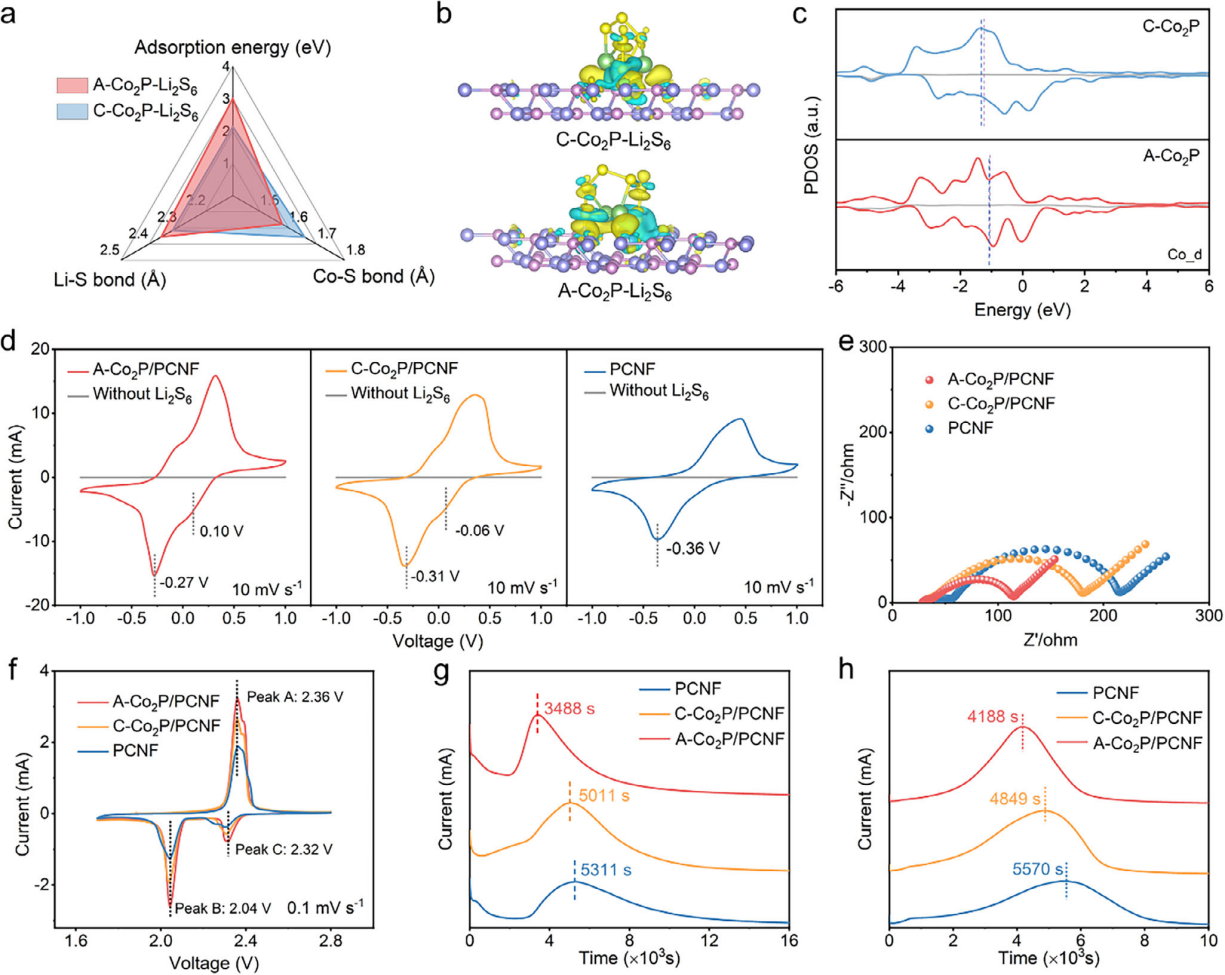
a) Spider chart of adsorption information of Li_2_S_6_ on A─Co_2_P and C─Co_2_P. b) Charge density differences of A─Co_2_P‐Li_2_S_6_ and C─Co_2_P‐Li_2_S_6_. The yellow region represents charge gain while the cyan region corresponds to charge loss. c) Co 3d PDOS of A─Co_2_P and C─Co_2_P. d) CV curves and e) EIS spectra of Li_2_S_6_ symmetric cells with different electrodes. f) CV curves of different electrodes. g) Li_2_S deposition and h) Li_2_S dissolution profiles.

To elucidate the influence of P vacancy and lattice distortion on the interfacial interaction between LiPSs and Co_2_P, density functional theory (DFT) calculations were performed. As shown in **Figure** **3**a, the binding energies of A─Co_2_P and C─Co_2_P to Li_2_S_6_ are −3.01 and −2.14 eV, respectively. The stronger interaction for the former originates from the unsaturated Co sites that strengthen the Co─S bonds and thus weaken the Li─S bonds in LiPSs, indicating that A─Co_2_P could more effectively inhibit the shuttle effect and enhance the conversion kinetics of LiPSs. This theoretical insight is further corroborated by experimental Li₂S₆ adsorption tests: after immersing A─Co₂P/PCNF and C─Co₂P/PCNF in a Li₂S₆ solution, the solution with A─Co₂P/PCNF shows a significantly lighter coloration, while the UV–Vis spectra demonstrate a stronger reduction in Li₂S₆ peak intensity for A─Co₂P/PCNF compared to C─Co₂P/PCNF and PCNF (Figure S5, Supporting Information), confirming its superior adsorption capability. In addition, the charge density difference was calculated to further visualize the electron redistribution of A─Co_2_P after interacting with LiPSs. Notably, a significant electron accumulation was observed along the connection between S and Co atoms (Figure 3b), indicating the presence of a strong interaction, especially for A─Co_2_P. It should be noted that the lack of electrons around Co atoms makes it more susceptible to nucleophilic attack and allows for electron coupling with the p‐orbital electrons of adsorbed LiPSs, ultimately promoting the dissociation of Li─S bonds.^[^ ^38^ ^]^ In addition, the partial density of states (pDOS) profiles of Co 3d orbitals were further simulated, as shown in Figure 3c. It reveals that both C─Co_2_P and A─Co_2_P exhibit metallic characteristics, with the *d*‐band center of A─Co_2_P closer to the Fermi level in comparison with that of C─Co_2_P. According to the *d*‐band center theory,^[^ ^39^ ^]^ the increased *d*‐band center results in a higher energy level of the antibonding orbitals, which leads to a more stable electron filling and a higher adsorption strength.

To comprehensively assess the electrocatalytic effect of A─Co_2_P/PCNF for the LiPSs redox reaction, a series of electrokinetic characterizations were performed. Figure 3d shows the cyclic voltammetry (CV) profiles of the symmetric cells assembled with identical electrodes using Li_2_S_6_ electrolyte at a scan rate of 10 mV s^−1^. Compared to the symmetric cells without Li_2_S_6_, all the symmetric cells with Li_2_S_6_ exhibit obvious current response, manifesting that the double‐layer capacitance has negligible contribution to the overall current, and the current response is mainly attributed to the redox reactions of Li_2_S_6_. Furthermore, A─Co_2_P/PCNF exhibits sharper redox peaks and smaller voltage polarization compared to C─Co_2_P/PCNF and PCNF, indicating that A─Co_2_P can effectively enhance the LiPSs redox kinetics toward the liquid‐liquid conversion.^[^ ^40^ ^]^ Meanwhile, the electrochemical impedance spectroscopy (EIS) curves of symmetrical cells with different electrodes (Figure 3e) show that the semicircle of A─Co_2_P/PCNF is smaller than that of C─Co_2_P/PCNF and PCNF, suggesting the lower charge transfer resistance and prompted interfacial charge transfer kinetics for Li_2_S_6_ redox reaction by A─Co_2_P/PCNF.^[^ ^41^ ^]^ Furthermore, CV tests were carried out to investigate the redox kinetics of Li─S batteries (Figure 3f). The two typical cathodic peaks represent the successive reduction processes of S_8_ to Li_2_S_x_ (4 ≤ x ≤ 6) (Peak C) and then to Li_2_S/Li_2_S_2_ (Peak B), respectively, while the anodic peak corresponds to the oxidation process of Li_2_S/Li_2_S_2_ (Peak A).^[^ ^42^ ^]^ Notably, A─Co₂P/PCNF delivers the highest peak current and the smallest electrochemical polarization (≈0.32 V), demonstrating superior sulfur redox kinetics. In contrast, C─Co₂P/PCNF and PCNF exhibit larger polarization gaps of ≈0.36 V and ≈0.39 V, respectively, consistent with their relatively lower catalytic activities. Moreover, to quantify the catalytic ability of different materials toward different LiPSs conversion reactions, corresponding Tafel plots are demonstrated in Figure S6 (Supporting Information). It is apparent that A─Co_2_P/PCNF shows the smallest Tafel slope for both sulfur reduction (40 mV dec^−1^) and oxidation (44 mV dec^−1^) reactions compared to the counterparts, suggesting its strongest capability for enhancing the electrocatalytic conversion of sulfur species because the unsaturated Co sites allow a stronger LiPSs adsorption strength to promote the breakage of Li─S bonds in the LiPSs chains.

It should be mentioned that fast Li^+^ transfer kinetics at the LiPSs/A─Co_2_P/PCNF interface also counts for the catalytic conversion of sulfur species. To further evaluate the Li^+^ diffusion kinetics for different electrodes, CV measurements at different scan rates from 0.1 to 0.5 mV s^−1^ were conducted (Figure S7a–c, Supporting Information). It can be observed that peak A moves toward higher potentials while peak B and peak C move toward lower potentials by increasing the scan rate for all three electrodes. The relationship between the peak current and the square root of the scan rate for each electrode is shown in Figure S7d–f (Supporting Information). According to the Randles–Sevcik equation,^[^ ^43^ ^]^ A─Co_2_P/PCNF exhibits a larger Li^+^ diffusion coefficient for both the reduction and oxidation processes of LiPSs (Figure S8, Supporting Information), indicating a smaller Li^+^ diffusion barrier on A─Co_2_P/PCNF. Benefiting from the promoted LiPSs conversion kinetics, the Li_2_S deposition and dissolution processes, which exhibit the lowest reaction kinetics but contribute the highest capacity, are also facilitated.^[^ ^44^ ^]^ As revealed in Figure 3g,h, the potentiostatic charge/discharge results demonstrate that A─Co_2_P/PCNF demonstrates the earliest response time and highest peak intensity for both Li_2_S deposition and dissolution processes, corroborating a more facilitated Li_2_S conversion process. Overall, these results clearly demonstrate the enhanced interfacial charge transfer kinetics and sulfur redox kinetics by A─Co_2_P/PCNF, which should be beneficial for the electrochemical performance of Li─S batteries.

**Figure 4:**
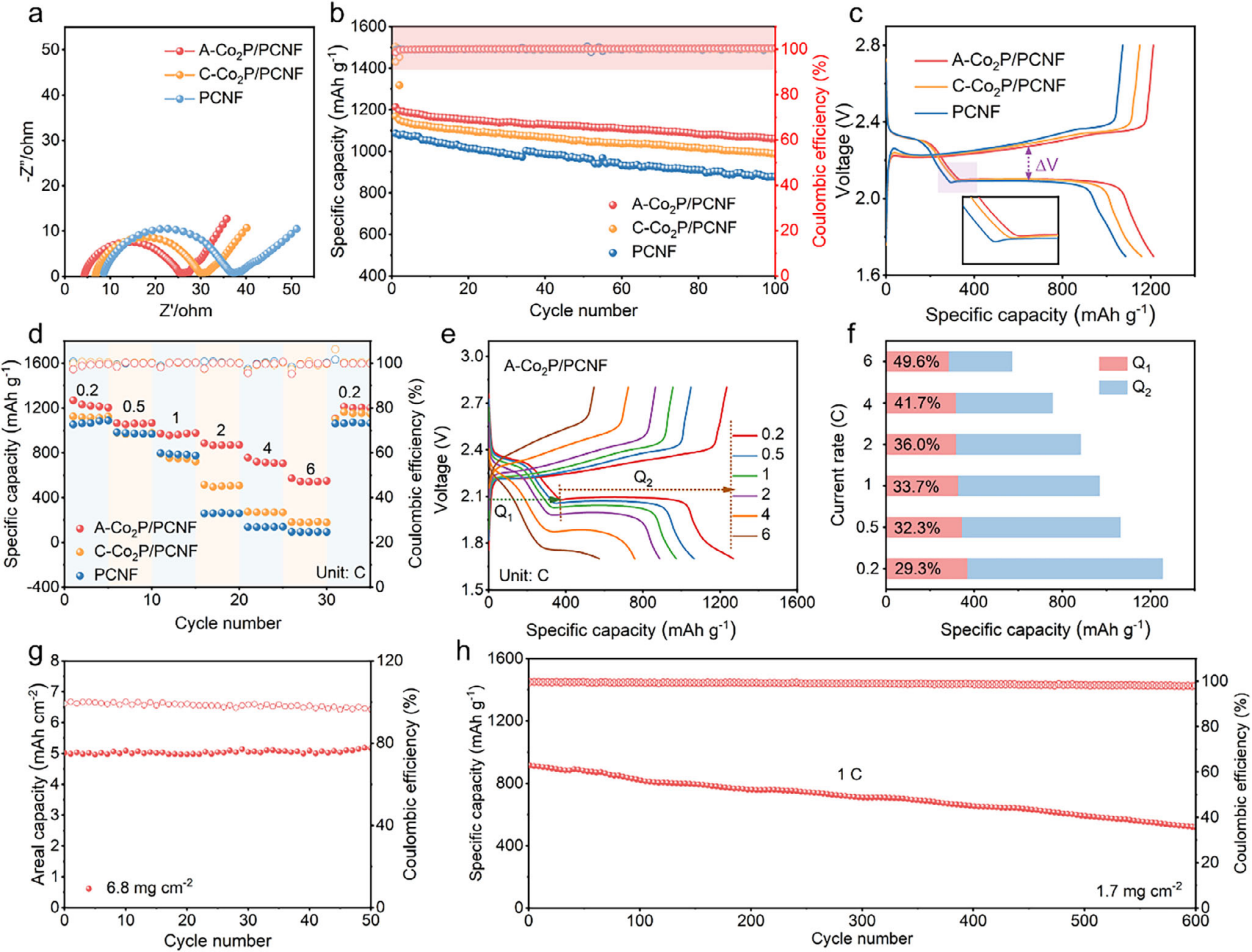
a) EIS spectra of different cathodes. b) Cycling performances and c) corresponding charge/discharge profiles. d) Rate performances. e) Charge/discharge profiles of A─Co_2_P/PCNF cathode at different rates. f) Discharge capacity from high plateau (denoted as Q_1_) and low plateau (denoted as Q_2_) at different rates. g) Cycling performance of A─Co_2_P/PCNF cathode with high sulfur loadings at 0.1 C. h) Long‐term cycling stability of A─Co_2_P/PCNF cathode at 1 C.

Therefore, electrochemical measurements were systematically carried out for the Li─S batteries assembled with A─Co_2_P/PCNF, C─Co_2_P/PCNF, and PCNF. Note that Li_2_S_6_ catholyte was used as the active material during the tests mainly because of the following two reasons: 1) The amount of active material can be accurately controlled by changing the concentration and amount of Li_2_S_6_ catholyte (10 µL of 1 m Li_2_S_6_ equals to 1.9 mg S) without the usage of toxic CS_2_ to dissolve S and the fussy heat‐melting process, 2) The liquid Li_2_S_6_ catholyte can spontaneously flow into the porous structures of A─Co_2_P/PCNF and avoid the electrolyte wetting process in the first cycle, which can efficiently promote the utilization of active materials.^[^ ^32^ ^]^ The corresponding EIS spectra (**Figure** **4**a) reveal that A─Co_2_P/PCNF exhibits a smaller semicircle diameter compared to that of C─Co_2_P/PCNF and PCNF, confirming the reduced charge transfer resistance during the battery cycling. Moreover, A─Co_2_P/PCNF delivers the highest cycling stability with an initial capacity of 1212 mAh g^−1^ and a capacity retention of 1061 mAh g^−1^ after 100 cycles at 0.2 C, which are superior to that of C─Co_2_P/PCNF and PCNF (Figure 4b). This enhanced capacity is largely attributed to the exceptional catalytic activity of A─Co_2_P/PCNF, as indicated by the minimal contribution from the material itself (Figure S9, Supporting Information). In addition, the corresponding voltage‐capacity curves all exhibit two discharge and one charge plateaus (Figure 4c), which correspond to the stepwise sulfur reduction and Li_2_S oxidation processes respectively, and match well with the CV results. Notably, a smaller voltage polarization between discharge and charge plateaus (ΔV) and lower overpotential for A─Co_2_P/PCNF suggest a kinetically favorable redox reaction of Li─S chemistry. The enhanced cycling performance clearly reveals the higher sulfur utilization and reaction efficiency enabled by A─Co_2_P/PCNF, which can be attributed to the establishment of a highly adsorptive barrier and promoted redox kinetic that inhibit the shuttling behaviors of LiPSs. The superiority of A─Co_2_P/PCNF is also reflected in the rate capability, which delivers the highest discharge capacities of 1268, 1065, 972, 887, 757, and 573 mAh g^−1^ at incremental current densities of 0.2, 0.5, 1, 2, 4, and 6 C, respectively (Figure 4d). When the current density reverts to 0.2 C, a high reversible capacity of 1102 mAh g^−1^ is still retained, accompanied by the typical voltage plateaus (Figure 4e). Despite the capacity fading and polarization rising with increasing the current rate, the second plateau corresponding to the transformation from Li_2_S_4_ to Li_2_S/Li_2_S_2_ still provides a considerable discharge capacity even at an elevated current rate of 6 C as a consequence of the accelerated redox reaction of LiPSs enabled by A─Co_2_P/PCNF (Figure 4f).

To further evaluate the potential of A─Co_2_P/PCNF for the practical application of Li─S batteries, the electrochemical performance under high sulfur loading conditions was also characterized (Figure 4g). With an elevated sulfur loading of 6.8 mg cm^−2^, a high reversible capacity of 5.2 mAh cm^−2^ is achieved at 0.1 C with a stable cycling for 50 cycles, suggesting that A─Co_2_P/PCNF can effectively confine and catalytically convert LiPSs to avoid the loss of active material even at high LiPSs concentrations because of the enhanced adsorption capability of A─Co_2_P/PCNF though amorphization strategy. In addition, long‐term cycling stability is another crucial parameter to evaluate the practical application of Li─S batteries. As shown in Figure 4h, a low capacity decay of 0.072% per cycle after 600 cycles at 1 C is realized for A─Co_2_P/PCNF, demonstrating the effective immobilization of LiPSs and remarkable redox electrocatalytic activity of A─Co_2_P/PCNF.

**Figure 5:**
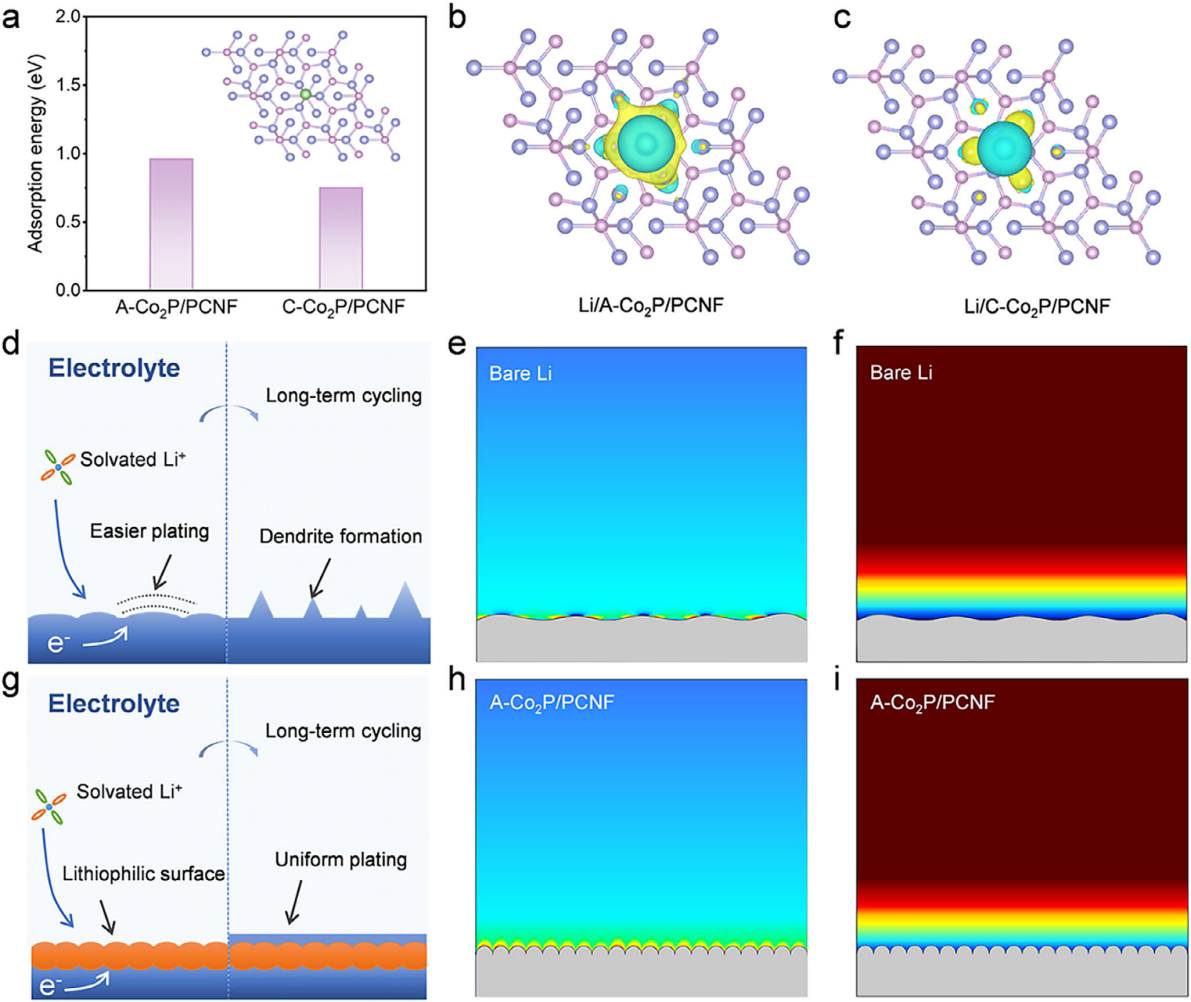
a) Binding energy of Li atom with A─Co_2_P/PCNF and C─Co_2_P/PCNF. Charge density difference patterns of b) A─Co_2_P/PCNF‐Li and c) C─Co_2_P/PCNF‐Li. d) Schematic illustration of Li deposition on bare Li and corresponding COMSOL simulations of e) current density distribution and f) Li^+^ flux. g) Schematic illustration of Li deposition on A─Co_2_P/PCNF‐Li and corresponding COMSOL simulations of h) current density distribution and i) Li^+^ flux. The color shift from red to blue corresponds to a change of electric field strength or Li concentration from high to low degree.

In addition to its role in stabilizing the sulfur cathode, the performance of Li anode is equally critical for the practical application of Li─S batteries. To elucidate the lithiophilic behavior of A‐Co₂P/PCNF, we conducted a comprehensive investigation by combining DFT calculations, charge density analysis, and phase‐field modeling. As shown in **Figure** **5**a, the binding energy between Li atoms and A‐Co₂P/PCNF (−0.96 eV) is considerably higher than that of C─Co₂P/PCNF (−0.75 eV), indicating stronger Li affinity. This enhanced interaction originates from the amorphous structure of Co₂P, which introduces unsaturated coordination sites and local lattice distortions. These structural features create electron‐deficient regions that strongly attract Li⁺ ions, facilitating uniform Li deposition and suppressing dendrite formation.^[^ ^45^ ^]^ Moreover, the charge density difference was calculated to understand the interaction between Li atoms and different substrates (Figure 5b,c), wherein the light blue and yellow colors correspond to charge depletion and accumulation, respectively. It is obvious that there is reduced charge density around Li atoms and strong electron density accumulation around Co_2_P, indicating that electrons are transferred from Li atoms to neighboring Co_2_P sites.^[^ ^22^ ^]^ The more intensive charge transfer between Li and A─Co_2_P/PCNF further confirms that A─Co_2_P/PCNF has a stronger interaction with Li atoms, which can provide sufficient nucleation sites for uniform Li deposition. Furthermore, phase‐field modeling of Li plating was carried out using COMSOL to understand the Li deposition process of different systems. For the bare Li metal, it suffers from severe Li dendrite formation and volumetric change during repeated stripping/plating cycles (Figure 5d). Because of the inhomogeneous distribution of the electric field, Li^+^ also tends to aggregate near the top of Li crystal tip and further accelerates the Li dendrite growth, leading to the uneven distribution of current density and the concentration polarization at the bulge of Li metal surface (Figure 5e,f). While for the Li metal coated with A─Co_2_P/PCNF, Li can uniformly deposit on the lithiophilic surface of A─Co_2_P/PCNF and remain stable after long‐term cycles (Figure 5g), which is strongly evidenced by the uniform distribution of current density and Li^+^ concentration near the A─Co_2_P/PCNF surface (Figure 5h,i). These results collectively demonstrate that the rationally designed A─Co_2_P/PCNF architecture effectively regulates Li^+^ flux and deposition kinetics, addressing two critical challenges in Li metal anodes: dendrite suppression and interfacial stability.

**Figure 6:**
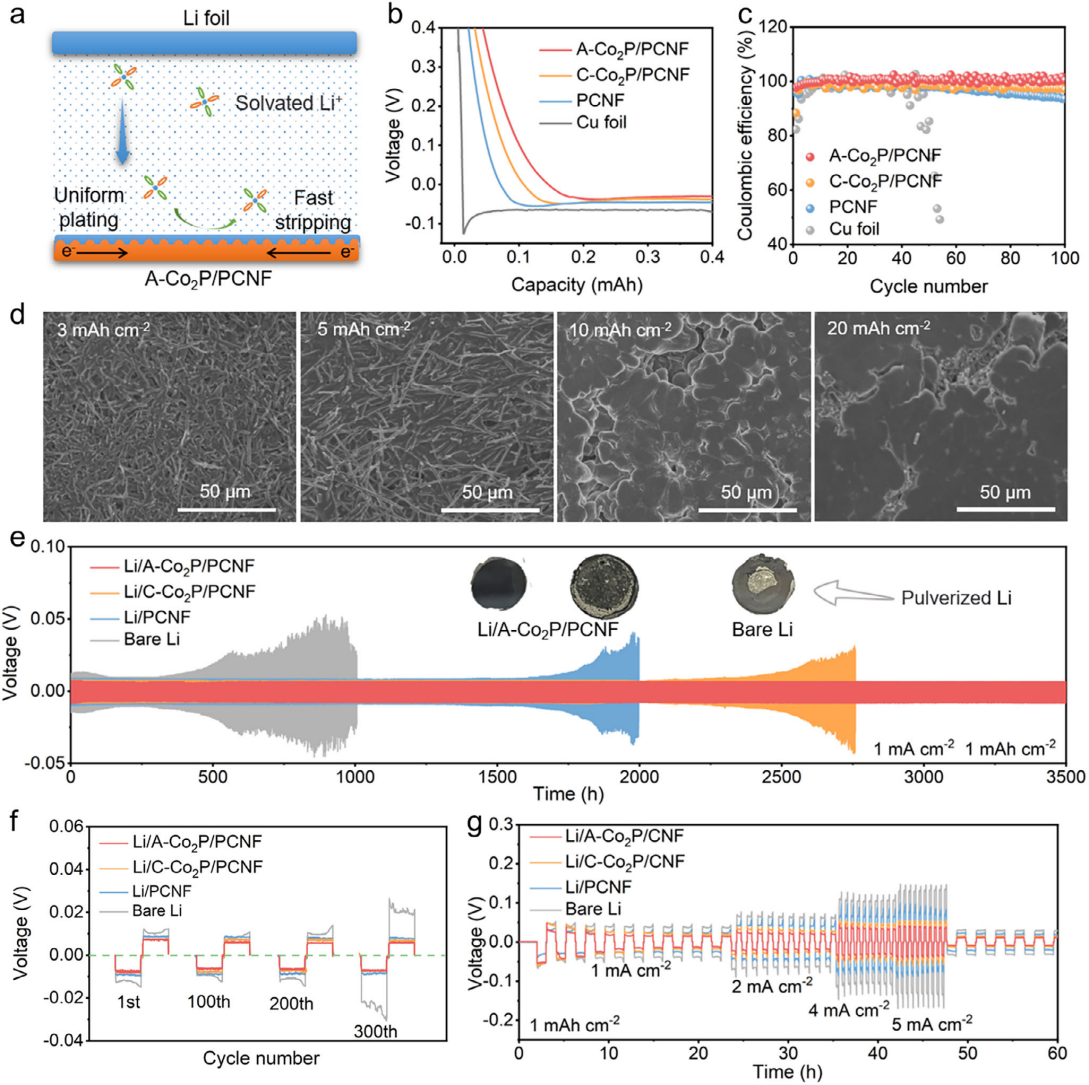
a) Schematic illustration of Li deposition on A─Co_2_P/PCNF. b) Voltage profiles of Li plating/stripping on different substrates at a current density of 1.0 mA cm^−2^ with a capacity of 1.0 mAh cm^−2^. c) CE at a current density of 1.0 mA cm^−2^ with a capacity of 1.0 mAh cm^−2^. d) SEM images of A─Co_2_P/PCNF deposited with different amount of Li. e) Galvanostatic cycling of symmetric cells at a current density of 1.0 mA cm^−2^ with a capacity of 1.0 mAh cm^−2^ and f) enlarged profiles at different cycles. g) Rate performances of symmetric cells with a fixed capacity of 1.0 mAh cm^−2^.

To experimentally elucidate the lithiophilic features of A─Co_2_P/PCNF, Li nucleation overpotential was comparatively evaluated with a two‐electrode configuration cell using A─Co_2_P/PCNF, C─Co_2_P/PCNF, PCNF, and Cu foil as the working electrode and Li metal as the counter electrode (**Figure** **6**a). As shown in Figure 6b, the voltage profile of Cu foil exhibits a sharp potential drop at a current density of 1.0 mA cm^−2^, while the voltage profiles for the electrodes with fibrous skeletons manifest rather smooth voltage dipping accompanied by much smaller overpotential, especially for that of A─Co_2_P/PCNF, suggesting that the amorphization of Co_2_P can considerably influence the Li nucleation behavior because of the regulated interaction with Li. In addition, A─Co_2_P/PCNF demonstrates the highest Coulombic efficiency (CE) over 100 cycles (≈99%) among all the investigated electrodes, suggesting the highly reversible Li plating/stripping behavior (Figure 6c). Furthermore, the surface morphology was investigated by SEM to clarify the Li plating behavior on A─Co_2_P/PCNF. As shown in Figure 6d and Figure S10 (Supporting Information), Li preferentially deposits along the carbon nanofiber skeleton under a relatively low plating capacity of 3 mAh cm^−2^ because of the lithiophilicity of A─Co_2_P/PCNF. Over the progressive plating process, the inherent interspaces of interlaced nanofibers are covered with Li with the formation of an island‐liked cluster morphology. When the plating capacity reaches to an ultrahigh amount of 20 mAh cm^−2^, the interfaces between clusters get blurred, and relatively smooth and dense Li layers with large grains are formed without the formation of Li dendrites.

The prepared nanofiber films were then posited on the top of Li anodes to form Li/A─Co_2_P/PCNF, Li/C─Co_2_P/PCNF, and Li/PCNF for the preparation of Li symmetric cells. Figure 6e compares the long‐term cycling behaviors of the Li symmetric cells with a stripping/plating capacity of 1 mAh cm^−2^ at a current density of 1 mA cm^−2^. Li/A─Co_2_P/PCNF exhibits consistently low voltage hysteresis and superior reversibility over 3500 h, in sharp contrast to that of Li/C─Co_2_P/PCNF, Li/PCNF, and bare Li, implying the remarkable suppressed growth of Li dendrites. When increasing the stripping/plating capacity to 3 mAh cm^−2^ at a current density of 3 mA cm^−2^ (Figure S11, Supporting Information), Li/A─Co_2_P/PCNF can still stably cycle for 500 h while the bare Li experiences a short circuit after 210 h. This showcases the effectiveness of incorporating a porous skeleton interlayer with lithiophilic A─Co_2_P sites in stabilizing the Li anode (Table S2, Supporting Information). Notably, the bare Li displays significant voltage hysteresis accompanied by high overpotential and dramatic voltage fluctuation (Figure 6f), which could be attributed to uncontrolled SEI breakage and uneven Li deposition (insert of Figure 6e; Figure S12, Supporting Information).^[^ ^46^ ^]^ The rate capabilities of the symmetric cells were further evaluated at varied current densities from 1 to 5 mA cm^−2^ with a capacity of 1 mAh cm^−2^ (Figure 6g). Clearly, Li/A─Co_2_P/PCNF exhibits the lowest overpotential at different current densities compared to other electrodes, manifesting the high potential of A─Co_2_P/PCNF for meeting the demand for high charge/discharge rates in practical Li─S batteries.

**Figure 7:**
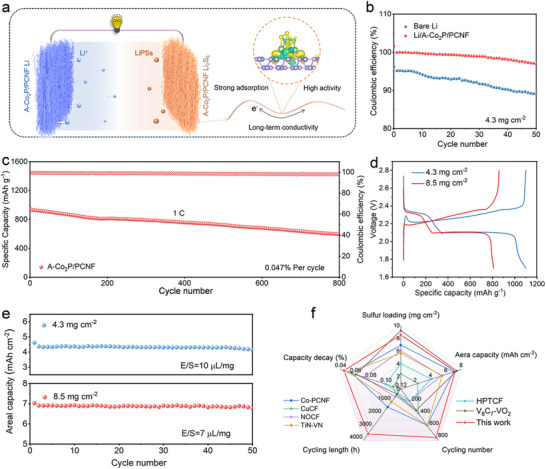
a) Schematic illustration of A─Co_2_P/PCNF dual‐functional fibrous scaffold‐enabled Li−S full batteries. b) CE profiles with bare Li and A─Co_2_P/PCNF‐Li. c) Long‐term cycling stability of Li−S full batteries. d) Charge/discharge profiles and e) cycling performance of Li−S full batteries with high sulfur loadings at 0.1 C. f) Spider chart of electrochemical performances enabled by A─Co_2_P/PCNF dual‐functional fibrous scaffold in comparison with the recently reported dual‐functional Li−S batteries.

To demonstrate the synergistic effect of LiPSs conversion and Li dendrite suppression enabled by A─Co_2_P/PCNF, the full batteries coupling Li_2_S_6_/A─Co_2_P/PCNF cathode and Li/A─Co_2_P/PCNF anode were assembled, as schematically illustrated in **Figure** **7**a. The CV curves of full batteries (Figure S13, Supporting Information) show two characteristic redox peaks matching those of half cells, confirming effective LiPSs conversion in the practical battery system. Notably, the A─Co_2_P/PCNF‐based battery exhibits a reduced polarization compared to its C─Co_2_P/PCNF counterpart, highlighting the superior redox kinetics enabled by the atomic‐level structural advantages of A─Co_2_P/PCNF. Figure 7b depicts the CE of Li−S batteries with bare Li and A─Co_2_P/PCNF‐Li. It is worth mentioning that the battery with bare Li anode shows a low CE and obvious decay, while for the full battery with A─Co_2_P/PCNF protected Li anode, the CE achieves near 100% and is more stable during cycling, demonstrating the significance of A─Co_2_P/PCNF in practical Li–S batteries. In addition, the full batteries with a sulfur loading of 1.7 mg cm^−2^ can obtain a high initial capacity of 915.1 mAh g^−1^, which still sustains a reversible capacity of 590.9 mAh g^−1^ after 800 cycles at 1 C with a low fading rate of 0.047% per cycle (Figure 7c), indicating the overwhelming cyclic stability. To further demonstrate the practical application of A─Co_2_P/PCNF, cycling performances of the full batteries with different sulfur loadings were measured at a current density of 0.1 C. As shown in Figure 7d, the charge/discharge profiles of full batteries with a high sulfur loading of 4.3 and 8.5 mg cm^−2^ show two discharge plateaus mentioned above, demonstrating the remarkable redox kinetics of LiPSs. With a sulfur loading of 4.3 mg cm^−2^, a high reversible capacity of 4.6 mAh cm^−2^ is achieved at 0.1 C with a stable cycling for 50 cycles. By increasing the sulfur loading to 8.5 mg cm^−2^ with a decreased E/S ratio of 7 µL mg^−1^, a high areal capacity of 6.6 mAh cm^−2^ is still obtained (Figure 7e), which is higher than the capacity of the half cell with bare Li anode shown in Figure 4g. The spider chart (Figure 7f; Tables S2 and S3, Supporting Information) compares the electrochemical performances of our batteries with the reported state‐of‐the‐art two‐in‐one Li─S systems. Benefitting from the synchronously protective effect of A─Co_2_P/PCNF on the sulfur cathode and Li anode, the assembled full batteries demonstrate very competitive advantages for practical application.

## 3 Conclusion

In summary, a dual‐functional carbon nanofiber skeleton implanted with A─Co_2_P is devised and employed as a stabilizer for high‐loading sulfur cathode and dendrite‐free Li anode in Li─S full batteries. It has been demonstrated that the amorphization strategy effectively increases the exposure of Co active sites in Co_2_P with undercoordinated sites and modulated *d*‐band center for enhanced adsorbability and catalytic activity toward LiPSs, thus largely inhibiting the shuttle effect of LiPSs. In addition, sufficient exposure to lithiophilic A─Co_2_P also promotes uniform Li deposition with suppressed formation of dendrites and enhanced stability. Meanwhile, the intact 1D carbon fibrous network facilitates excellent electronic and ionic conductivity and provides abundant space for uniform Li_2_S deposition and Li deposition during charge‐discharge cycling. Therefore, the constructed full Li‐S batteries deliver a superior specific capacity with enhanced cycle stability over 800 cycles, even under practical sulfur loading and electrolyte content conditions. Our present study provides a promising strategy to collectively regulate LiPSs catalysis and suppress Li dendrite growth for the development of high‐energy and durable Li─S batteries.

## Conflict of Interest

The authors declare no conflict of interest.

## Supporting information



Supporting Information

## Data Availability

The data that support the findings of this study are available from the corresponding author upon reasonable request.;
