# Supplementary material for: A Bifunctional Fibrous Scaffold Implanted with Amorphous Co2P as both Cathodic and Anodic Stabilizer for High‐Performance Li─S Batteries
Source: Adv Sci (Weinh). 2025 Apr 1;12(29):2501153. doi: 10.1002/advs.202501153 (PMC12362775; doi:10.1002/advs.202501153)
Supplement: Supplementary file 1 — Supporting Information [file ADVS-12-2501153-s001.docx]

**Supporting Information**

**A Bifunctional Fibrous Scaffold Implanted with Amorphous Co_2_P** **as** **Both** **Cathodic and Anodic Stabilizer for High-Performance Li−S Batteries**

Gang Zhao,^1, 2^ Tianran Yan,^1,*^ Lei Wang,^1^ Cheng Yuan,^1^ Tong Chen,^1^ Bin Wang,^3,*^ Chen Cheng,^1^ Pan Zeng,^4^ Yude Su,^2,*^ and Liang Zhang^1,5*^

1. Institute of Functional Nano & Soft Materials (FUNSOM), Soochow University, 199 Ren'ai Road, Suzhou 215123, Jiangsu, China
2. School of Chemistry and Materials Science, University of Science and Technology of China, Hefei, 230026, Anhui, China
3. China Minmetals Graphite Industry Co., Ltd. (Heilongjiang), Hegang 154100, Heilongjiang, China
4. Institute for Advanced Study, School of Mechanical Engineering, Chengdu University, 610106, Chengdu, China
5. Jiangsu Key Laboratory of Advanced Negative Carbon Technologies, Soochow University, Suzhou, 215123, Jiangsu, China

*Emails: liangzhang2019@suda.edu.cn; suyude@ustc.edu.cn; wangbin0502@163.com; tryan@suda.edu.cn

**Experimental Section**

**Preparation of** **ZnCo-ZIF Nanocrystals**

3.70 g of 2-methylimidazole (purity >98.0%, Aladdin) was dissolved in 80 mL of methanol (purity >99.5%, Shanghai Lingfeng Chemical Reagent) to form a clear solution and then mixed with 80 mL of methanol solution containing 1.606 g of Zn(NO_3_)_2_·6H_2_O (purity >99%, Aladdin) and 100 mg of Co(NO_3_)_2_·6H_2_O (purity >99.99%, Aladdin). After vigorous stirring for 1 h, the mixture was rested at room temperature with a standing time of 12 h. The resulting precipitate was collected by centrifugation and washed by methanol for three times. After drying in vacuum at 60 °C for 12 h, the final product with purple color was obtained. Bare ZIF nanocrystal was prepared by a similar procedure except for the addition of Co(NO_3_)_2_·6H_2_O.

**Preparation of A-Co_2_P/PCNF**

0.5g triphenylphosphine (TPP purity >95%, Aladdin 95%) was dissolved in 10 mL of dimethylformamide (DMF; purity >99.8%, Aladdin) firstly, and then1 g of as-synthesized ZnCo-ZIF was dispersed in the above solution by sonication for 1 h, followed by the addition of 1 g of polyacrylonitrile (PAN; M.W. ∼150 000, Aldrich). The precursor mixture was stirred for 12 h to ensure homogeneity and then loaded into a syringe equipped with a metal needle for electrospinning. The electrospinning process was conducted under controlled environmental conditions, with the relative humidity maintained at approximately 40%. The distance between the needle tip and the aluminum foil collector was set to 15 cm, and the spinning solution was fed at a rate of 1 mL h⁻¹. A positive voltage of 14 kV was applied to facilitate fiber formation. The resulting fibrous film was subjected to a two-step thermal treatment: pre-oxidation in air at 220 °C for 2 h with a heating rate of 1 °C min⁻¹ followed by carbonization at 950 °C for 2 h in a nitrogen atmosphere at a heating rate of 5 °C min⁻¹. These optimized parameters ensured the production of uniform and mechanically robust nanofibers suitable for further applications.

**Preparation of** **C-Co_2_P/PCNF and PCNF**

Co/PCNF was prepared following the above method except the addition of TPP. Subsequently, Co/PCNF precursor was put at the downstream of furnace and ten times weight of NaH_2_PO_2_·H_2_O was put at the upstream of the furnace, respectively. The powders were annealed in N_2_ at 300 °C for 2 h and then C-Co_2_P/PCNF was obtained. For comparison, PCNF was obtained by substituting ZnCo-ZIF with bare Zn-ZIF in the electrospinning solution.

**Material Characterizations**

The morphologies were characterized by scanning electron microscopy (SEM, G500 ZEISS). The internal structure and high-resolution lattice were measured by transmission electron microscopy (TEM, 200X, Talos) equipped with energy dispersive X-ray spectrometer (EDS). X-ray diffraction (XRD) with Cu Kα radiation was performed at Empyrean in the range of 2θ = 5-90°. UV-vis spectroscopy was performed using UV-vis spectrophotometer (PE750). The Raman spectroscopy was recorded by the Raman spectrometer (Horiba HR800) with a 633 nm laser. The N_2_ adsorption/desorption isotherm was measured on a BSD-660M A6M to analyze the specific surface area, pore volume, and pore size distribution. The Co K-edge X-ray absorption fine structure (XAFS) measurement was performed at beamline 1W1B of Beijing Synchrotron Radiation Facility (BSRF) and beamline 11B of Shanghai Synchrotron Radiation Facility (SSRF). For ex-situ characterizations, standard procedures were used to handle samples, including the disassembly, rinse, and transfer. The electrodes at different amounts of Li plating were disassembled in the Ar filled glovebox (H_2_O < 0.1ppm, O_2_ < 0.1ppm) and rinsed with DME solution thoroughly to remove the surface residue. Then the electrodes were sealed for the following measurements.

**Li_2_S_6_ Adsorption Experiments**

The Li_2_S_6_ solution (2 mM) was prepared by dissolving sulfur and lithium sulfide (Li_2_S; purity >99.9%; Alfa) in the 1,2-dimethoxyethane (DME; purity >99.99%; Aladdin) solvent with a molar ratio of 5:1, followed by vigorous stirring for 24 h at 60 °C. Then 10 mg of PCNF, A-Co₂P/PCNF and C-Co₂P/PCNF powders were dispersed in 3 mL Li_2_S_6_ solution, respectively. After soaking for certain time, the supernatant was absorbed for UV-vis test.

**Nucleation and Dissolution of Li_2_S Measurements**

0.2 M Li_2_S_8_ solution was prepared by mixing sulfur and Li_2_S in tetraglyme (TEGDME; purity >99.5%; Macklin) solvent with a molar ratio of 7:1. The A-Co_2_P/PCNF, C-Co_2_P/PCNF and PCNF carbon matrix were directly used as self-standing cathodes and lithium foil is used as the anode, with 15 µl of 0.2 M Li_2_S_8_ solution as active materials. The assembled batteries were galvanostatically discharged to 2.06 V at a current of 0.112 mA and then potentiostatically discharged to 2.05 V until the discharged current was below 0.01 mA. Similarly, the batteries were assembled using the same electrode material and electrolyte. The batteries were first galvanostatically discharged to 1.7 V at a current of 0.112 mA and then galvanostatically discharged to 1.8 V at 0.1 mA to ensure that polysulfides were fully converted into solid Li_2_S. Then, the batteries were potentiostatically charged at 2.35 V until the charge current is below 0.01 mA for completely dissolving Li_2_S. Finally, the nucleation and dissolution capacity of Li_2_S were evaluated by calculating the integral area of the curve drawn according to Faraday's Law.

**Assembly of Li_2_S_6_ Symmetric Cells**

Identical electrodes (A-Co_2_P/PCNF, C-Co_2_P/PCNF and PCNF) were assembled into CR2032 coin cells with 20 μl Li_2_S_6_ electrolyte (0.2 M) in each cell. CV tests were performed on a CHI660E electrochemical workstation at 10 mV s^-1^ within the potential range of -1 to 1 V.

**Lithium Metal Anode Tests.**

All the coin cells were assembled in an Ar-filled glovebox with O_2_ and H_2_O content below 0.1 ppm. The coulombic efficiency test was evaluated in the coin-type cell (CR2032) with Li foil as the counter electrode and free-standing A-Co_2_P/PCNF, C-Co_2_P/PCNF, PCNF or Cu foil as working electrodes at a current density of 1.0 mA cm^-2^ with a capacity of 1.0 mAh cm^-2^. Galvanostatic cycling with different current densities and capacities in symmetric cells were conducted to evaluate the long-time cycling stability. Typically, the Li/A-Co_2_P/PCNF, Li/C-Co_2_P/PCNF and Li/PCNF electrodes were assembled by coating the membranes on the Li foil. In all measurements, Celgard 2500 was used as the separators.

**Assembly of Li-S Batteries** **and Electrochemical Measurements**

The obtained fibrous membrane was cut into discs with a diameter of 12 mm which were directly used as the self-standing cathode. The CR2032-type coin cells were assembled using lithium foil with diameter of 16 mm as anode and Celgard 2500 as separator in Ar-filled glovebox (O_2_ < 0.1 ppm, H_2_O < 0.1 ppm). The electrolyte consists of 1 M bis (trifluoromethane sulfonyl) imide (LiTFSI) in 1, 2-dimethoxyethane (DME) and 1, 3-dioxolane (DOL) (1:1 in volume) with 2.0 wt% LiNO_3_ as additive. The 1 M Li_2_S_6_-contained electrolyte was used as catholyte (10 μl of catholyte equals to 1.92 mg sulfur), while certain amount of additional bare electrolyte was added to regulate the electrolyte-to-sulfur (E/S) ratio. Typically, 10 μl of catholyte and 40 μl of additional bare electrolyte with an areal sulfur loading of 1.7 mg cm^-2^ were used for rate performance and cyclability tests. For high sulfur loading tests, 25, 50 and 100 μl of catholyte were used to achieve the sulfur loading of 4.3, 8.5 and 17 mg cm^-2^, respectively. The galvanostatic charge-discharge test was measured by the LAND CT2001A battery test system. CHI660E electrochemical workstation was used for CV test and the scan rate was from 1 to 5 mV s^-1^ in the voltage of 1.7 to 2.8 V. Electrochemical impedance spectroscopy was measured in the frequency range of 10 mHz to 100 kHz.

**Full Li−S Cells Test**

Full cells were assembled using A-Co_2_P/PCNF as the cathode with Li_2_S_6_ catholyte as active materials and Li/A-Co_2_P/PCNF as the anode. Li foil with a thickness of 0.45 mm was employed. The electrolyte was 1 M LiTFSI in DME/DOL (volume ratio was 1:1) with 0.2 M LiNO_3_. The sulfur loading of the A-Co_2_P/PCNF was controlled by adding different amount of Li_2_S_6_ catholyte. Galvanostatic discharge/charge behaviors were investigated between 1.7 and 2.8 V (vs Li/Li^+^).

**COMSOL Multiphysics Simulations**

The COMSOL Multiphysics simulations were performed by Multiphysics 5.5 software based on lithium battery module simulation. The surface diffusion coefficient is 2×10^-7^ m^2^s^-1^. The Li ion diffusion coefficient in the electrolyte is 2×10^-9^ m^2^ s^-1^, the Li ion concentration is 1×10^3^ mol m^-3^, and the current density is 400 mA cm^-2^.

**DFT Calculations**

The density functional theory (DFT) calculations were carried out with the VASP code. The Perdew–Burke–Ernzerhof (PBE) functional within generalized gradient approximation (GGA) was used to process the exchange–correlation, while the projectoraugmented-wave pseudopotential (PAW) was applied with a kinetic energy cut-off of 500 eV, which was utilized to describe the expansion of the electronic eigenfunctions. The vacuum thickness was set to be 20 Å to minimize interlayer interactions. The Brillouin-zone integration was sampled by a Γ-centered 8 × 8 × 1 Monkhorst–Pack k-point. All atomic positions were fully relaxed until energy and force reached a tolerance of 1 × 10^-5^ eV and 0.03 eV/Å, respectively. The dispersion corrected DFT-D method was employed to consider the long-range interactions.

The adsorption energy (E_ads_) of a complex formed between two molecules, A and B, can be calculated using the following equation:

E_ads_ = E_complex_ - (E_A_ + E_B_)

Where: E_complex_ is the total energy of the molecular complex of A and B.

E_A_ and E_B_ are the total energies of isolated molecules A and B, respectively.


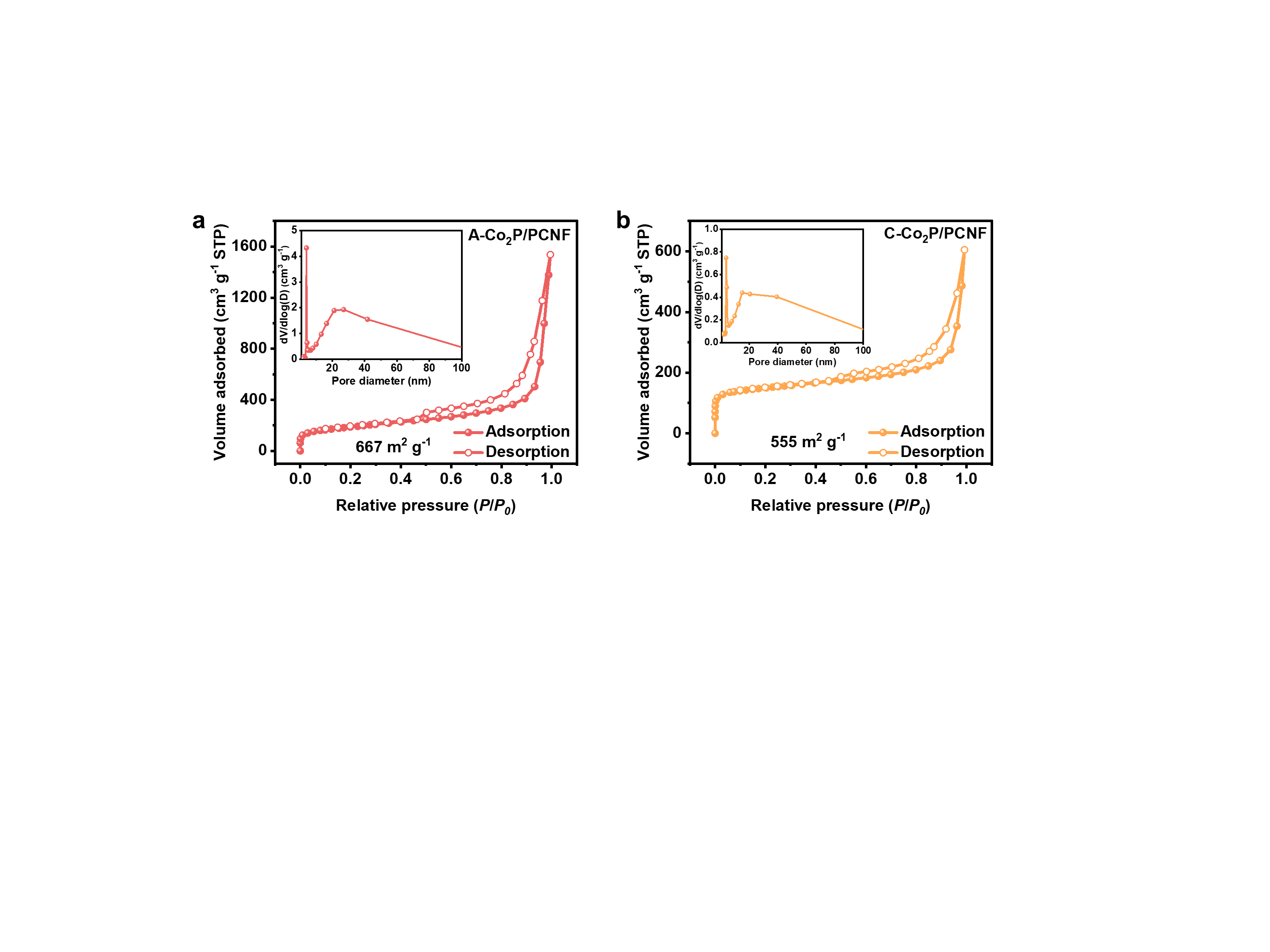


Figure S1. Nitrogen adsorption-desorption isotherms of A-Co₂P/PCNF and C-Co₂P/PCNF. The insets show the corresponding pore size distribution curve.


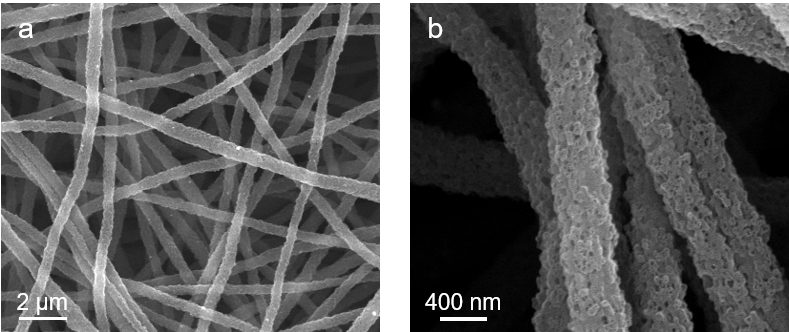


Figure S2. (a) Low- and (b) high-magnification SEM images of C-Co_2_P/PCNF.


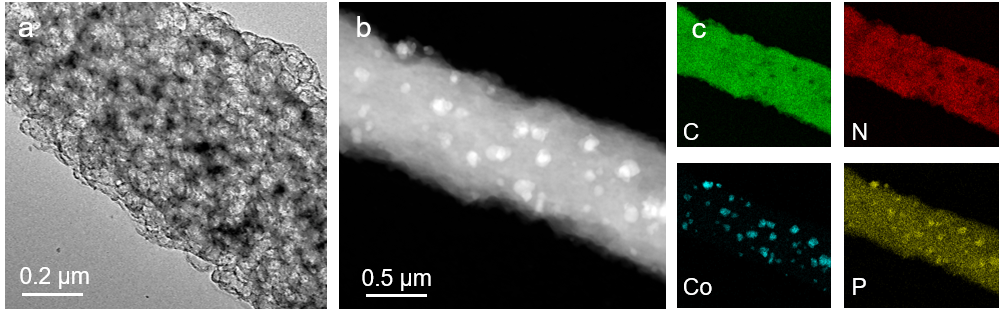


Figure S3. (a) TEM image of C-Co_2_P/PCNF. (b) HAADF-STEM image and (c) corresponding elemental maps of C-Co_2_P/PCNF.


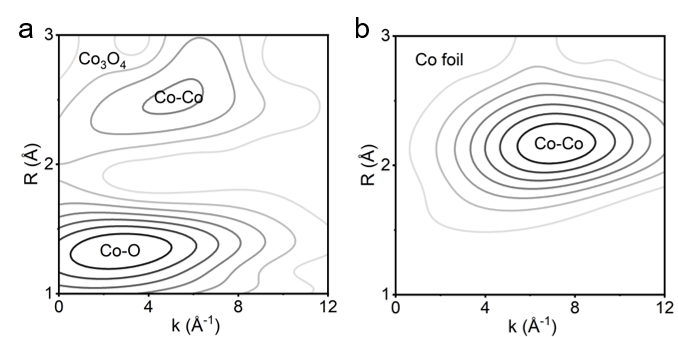


Figure S4. WT-EXAFS plots of (a) Co_3_O_4_ and (b) Co foil.


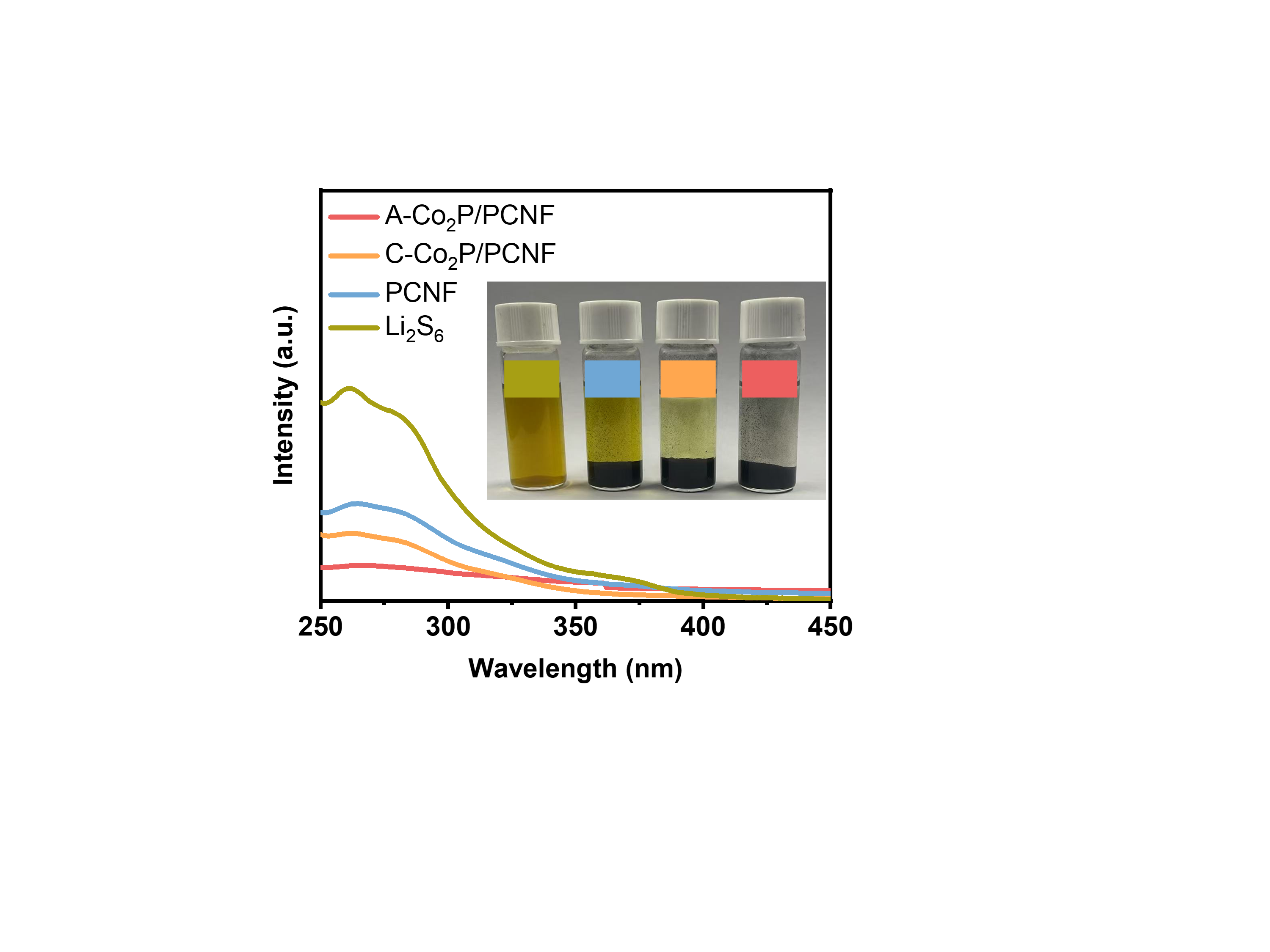


Figure S5. UV-vis spectra of Li_2_S_6_ solution before and after the addition of PCNF, C-Co₂P/PCNF and A-Co₂P/PCNF, inset is the digital picture of the adsorption of Li_2_S_6_ by PCNF, C-Co₂P/PCNF and A-Co₂P/PCNF.


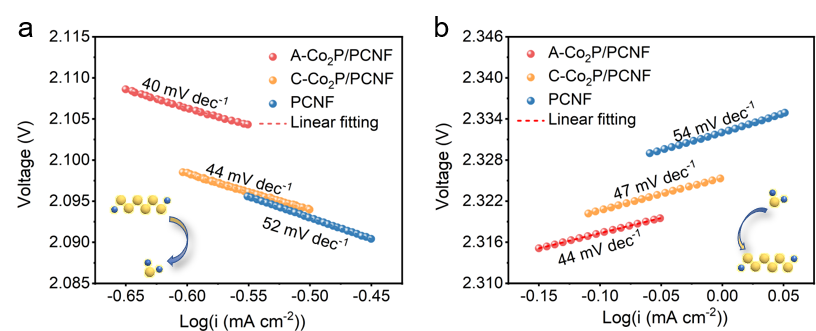


Figure S6. Tafel plots for (a) liquid-solid reaction and (b) solid-liquid reaction.


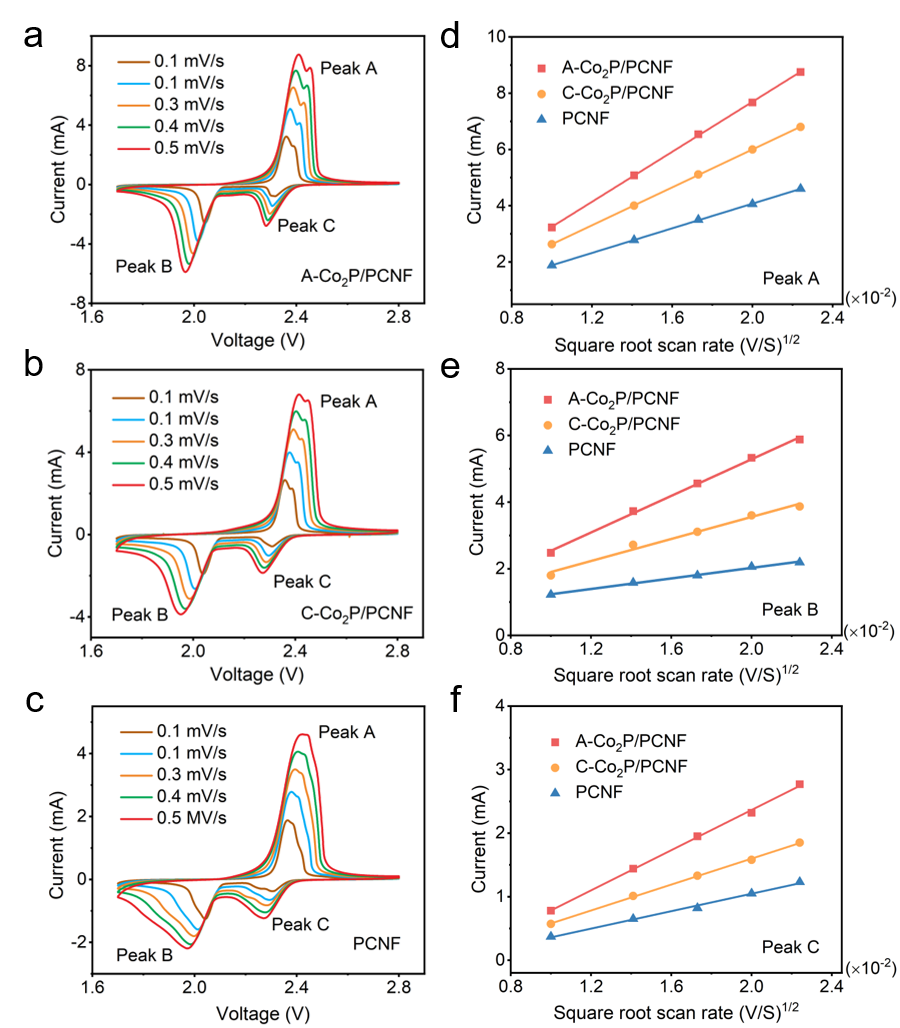


Figure S7. CV curves of Li–S batteries with (a) A-Co_2_P/PCNF, (b) C-Co_2_P/PCNF, (c) PCNF at different scan rates from 0.1 to 0.5 mVs^−1^ and (d-f) corresponding linear fits of redox peak currents versus the square root of scan rate.


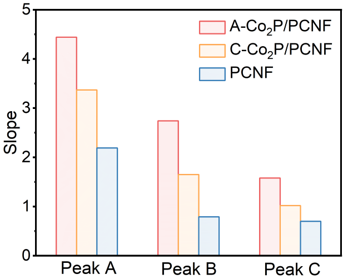


Figure S8. Slopes of linear fits for different cathodes.

Figure S9. Cycling performance of the battery with bare A-Co₂P/PCNF electrode.


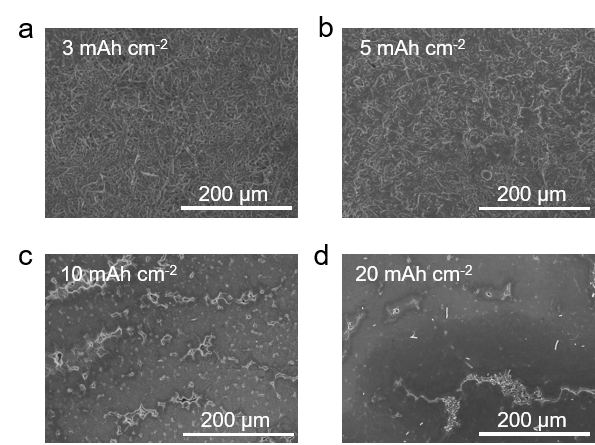


Figure S10. SEM images of A-Co_2_P/PCNF deposited with (a) 3 mAh cm^-2^, (b) 5 mAh cm^-2^, (c) 10 mAh cm^-2^ and (d) 20 mAh cm^-2^ of Li.


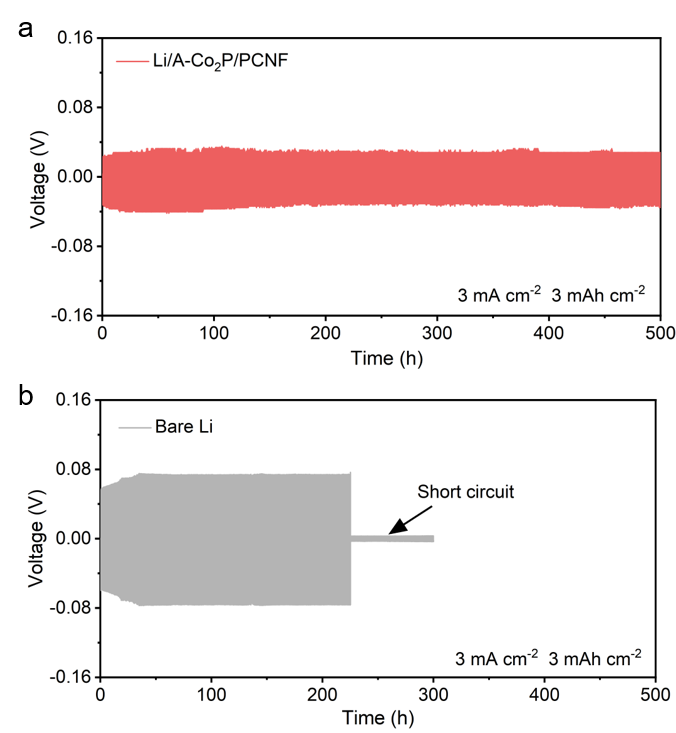


Figure S11. Cycling performances of (a) Li/A-Co_2_P/PCNF and (b) bare Li symmetric cells with a stripping/plating capacity of 3 mAh cm^-2^ at a current density of 3 mA cm^-2^.


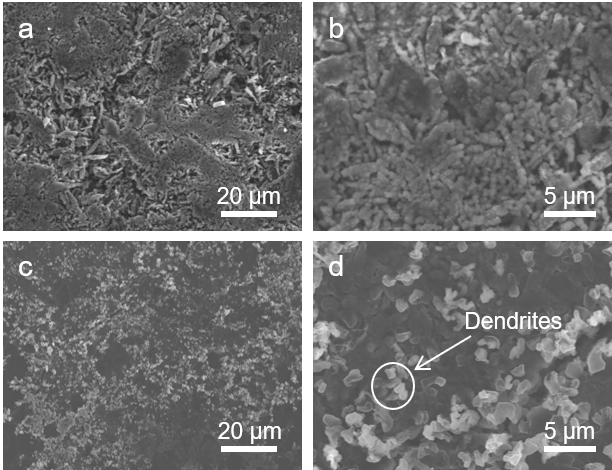


Figure S12. (a) and (b) SEM images of A-Co_2_P/PCNF after cycling. (c) and (d) SEM images of Li anode after cycling.

Figure S13. CV curves of full batteries with scan rate of 0.1 mV s^−1^.

**Table S1.** EXAFS fitting parameters at the Co K-edge.

| Sample | Path | R (Å) | ΔE (eV) | CN | ΔR (Å) | σ^2^  (10^-3^*Å^-2^） | R-factor |
| --- | --- | --- | --- | --- | --- | --- | --- |
| A-Co_2_P  /PCNF | Co-P | 2.167 | 7.87 | 3.83 | 0.08 | 9 | 0.02 |
|  | Co-Co | 2.566 |  | 1.96 | -0.02 | 15 |  |
| C-Co_2_P/  PCNF | Co-P | 2.167 | 0.93 | 4.12 | 0.01 | 6 | 0.04 |
|  | Co-Co | 2.566 |  | 1.83 | -0.04 | 16 |  |

Notes: *N* is coordination numbers, *R* is bond distance, σ^2^ is Debye-Waller factors, Δ*E*_0_ is the inner potential correction, and *R* factor is goodness of fitting.

Table S2. Comparison of electrochemical performances of Li anode employing different conductive materials.

|  | **Materials** | **Current density (mA cm^-2^)/Areal capacity**  **（mAh cm^-2^)** | **Lifespan (h)** | **References** |
| --- | --- | --- | --- | --- |
|  | A-Co_2_P/PCNF | 1/1  3/3 | 3500  500 | This work |
| Interlayers | Co-PCNF | 3/3 | 1500 | 1 |
|  | V_2_C MXene | 1/1 | 500 | 2 |
|  | 3DIO FCSe-QDs | 1/1 | 1400 | 3 |
|  | V_8_C_7_–VO_2_ | 3/1 | 370 | 4 |
|  | MCG@Cu | 1/3 | 1000 | 5 |
| Li hosts | TiN-VN@CNFs | 2/1 | 1000 | 6 |
|  | D-Cu@CuSe | 1/1 | 1800 | 7 |
|  | CuCF | 2/1 | 500 | 8 |
|  | CoSe@CCM | 1/1 | 600 | 9 |

| **Materials** | **Specific capacity**  **(mAh g^-1^)** | | **Cycle number** | **Rate/Decay**  **rate** | **References** |
| --- | --- | --- | --- | --- | --- |
| A-Co_2_P/PCNF | | 943 | 800 | 1 C/0.047% | This work |
| Co-PCNF | | ~960 | 600 | 0.2C/0.082% | 1 |
| V_2_C MXene | | 840 | 600 | 1 C/0.041% | 2 |
| TiN-VN@CNFs | | 870 | 600 | 2 C/0.051% | 6 |
| V_8_C_7_–VO_2_ | | 643 | 900 | 4 C/0.061% | 4 |
| D-Cu@CuSe | | 150 | 200 | 1 C/- | 7 |
| Co/N-PCNSs | | 850 | 200 | 1 C/0.12% | 10 |
| CC@CoP/C | | 800 | 600 | 2 C/0.016% | 6 |
| CuCF | | 938 | 260 | ~0.85 C/0.11% | 8 |
| CoSe@BNCNTs/CC | | 1051 | 800 | 0.5 C/0.066% | 11 |
| HE-PBA | | 590 | 850 | 1 C/0.05% | 12 |
| HPTCF | | ~1000 | 200 | 0.2 C/0.061% | 13 |
| NOCF | | ~910 | 200 | ~0.2 C/0.071% | 14 |

Table S3. Comparison of electrochemical performances of Li–S batteries employing “two-in-one” hosts.

**References**

1. Huang, T.; Y. Sun; J. Wu; J. Jin; C. Wei; Z. Shi; M. Wang; J. Cai; X. T. An; P. Wang; C. Su; Y. Y. Li; J. Sun, *ACS Nano* **2021,** *15*, 14105-14115.

2. Chen, L.; Y. Sun; X. Wei; L. Song; G. Tao; X. Cao; D. Wang; G. Zhou; Y. Song, *Adv. Mater.* **2023,** *35*, e2300771.

3. Huang, Y.; L. Lin; Y. Zhang; L. Liu; B. Sa; J. Lin; L. Wang; D. L. Peng; Q. Xie, *Nano-micro Lett.* **2023,** *15*, 67.

4. Cai, J.; J. Jin; Z. Fan; C. Li; Z. Shi; J. Sun; Z. Liu, *Adv. Mater.* **2020,** *32*, 2005967.

5. Wang, P.; B. Xi; Z. Zhang; N. Song; W. Chen; J. Feng; S. Xiong, *Small* **2021,** *17*, e2103744.

6. Yao, Y.; H. Wang; H. Yang; S. Zeng; R. Xu; F. Liu; P. Shi; Y. Feng; K. Wang; W. Yang; X. Wu; W. Luo; Y. Yu, *Adv. Mater.* **2020,** *32*, e1905658.

7. Shi, Z.; Z. Sun; X. Yang; C. Lu; S. Li; X. Yu; Y. Ding; T. Huang; J. Sun, *Small Sci.* **2022,** *2*, 2100110.

8. Chang, J.; J. Shang; Y. Sun; L. K. Ono; D. Wang; Z. Ma; Q. Huang; D. Chen; G. Liu; Y. Cui; Y. Qi; Z. Zheng, *Nat. Commun.* **2018,** *9*, 4480.

9. Hou, R.; Y. Li; Z. Wang; Z. Shi; N. Li; F. Miao; G. Shao; P. Zhang, *Small* **2023**, e2300868.

10. Liu, S.; J. Li; X. Yan; Q. Su; Y. Lu; J. Qiu; Z. Wang; X. Lin; J. Huang; R. Liu; B. Zheng; L. Chen; R. Fu; D. Wu, *Adv. Mater.* **2018,** *30*, 1706895.

11. Li, Y.; X. Wang; M. Sun; J. Xiao; B. Zhang; L. Ai; Z. Zhao; J. Qiu, *ACS Nano* **2022,** *16*, 17008-17020.

12. Shen, N.; T. Li; B. Li; Y. Wang; H. Liu; C. Guo; X. Chen; J. Li, *Nanoscale* **2024,** *16*, 7634-7644.

13. Cai, W.; G. Li; D. Luo; G. Xiao; S. Zhu; Y. Zhao; Z. Chen; Y. Zhu; Y. Qian, *Adv. Energy Mater.* **2018,** *8*, 1802561.

14. An, Y.; C. Luo; D. Yao; S. Wen; P. Zheng; S. Chi; Y. Yang; J. Chang; Y. Deng; C. Wang, *Nano-micro Lett.* **2021,** *13*, 84.
